# Electric Field Effects on Amine Regeneration in Post-Combustion Carbon Capture—Part I: Static Electric Fields as a Reference Mechanistic Baseline

**DOI:** 10.3390/molecules31091422

**Published:** 2026-04-25

**Authors:** Nasser D. Afify, Xianfeng Fan, Martin B. Sweatman

**Affiliations:** Institute for Materials and Processes, School of Engineering, The University of Edinburgh, Edinburgh EH9 3FB, UK; x.fan@ed.ac.uk (X.F.); martin.sweatman@ed.ac.uk (M.B.S.)

**Keywords:** post-combustion carbon capture, amine regeneration, quantum chemical calculations, density functional theory, infrared laser excitation, non-thermal laser effects

## Abstract

Although amine-based post-combustion carbon capture is among the most established routes for CO_2_ capture, it suffers from the high energy demand associated with amine regeneration. Recent research proposals suggest that microwave or frequency-tuned infrared heating may lead to more efficient amine regeneration processes. However, such approaches inherently introduce oscillating electromagnetic fields whose non-thermal effects on reaction pathways and energetics remain poorly understood. In this series paper, we employ high-accuracy quantum computational chemistry calculations to quantify the non-thermal effects of external electric fields on CO_2_ absorption and desorption in monoethanolamine (MEA) and triethanolamine (TEA) under both aqueous and non-aqueous conditions. In this first part, we focus on static electric fields in order to establish a mechanistic reference framework helpful for interpreting non-thermal effects arising from frequency-tuned infrared laser excitation, which are addressed in Part II of this series. Our results show that static electric fields stabilize CO_2_–amine reaction products, lowering absorption barriers, while consistently increasing both activation energies and reaction enthalpies associated with the amine regeneration process. This effect is particularly pronounced for MEA, where carbamate species become progressively more resistant to conversion to zwitterion as the field strength increases. These findings demonstrate that non-thermal static electric field effects counter the fundamental requirement for low-energy amine regeneration. By defining this intrinsic mechanistic limitation, the present study provides a useful baseline for assessing infrared laser-assisted carbon capture and underscores the importance of carefully selecting excitation frequencies to avoid adverse non-thermal stabilization effects.

## 1. Introduction

Amine-based post-combustion carbon capture (PCCC) technology is among the most mature and widely deployed approaches in the global effort to mitigate climate change [1]. This technology operates through a continuous cyclic process in which CO_2_ molecules are absorbed and subsequently desorbed by an amine solution [2,3]. An exhaust gas containing CO_2_ enters the bottom of an absorption column, where it comes into contact with the amine solution. The CO_2_ molecules react with primary amines, such as MEA, to form carbamate species at temperatures typically between 40 °C and 60 °C under atmospheric pressure [2,3]. The resulting CO_2_-rich amine solution then flows to a stripping column, where an endothermic regeneration reaction restores the spent amine and releases a purified CO_2_ stream for storage. The regenerated amine solution exits the bottom of the stripping column and is recirculated to the absorption column, thereby completing the absorption–desorption cycle.

Despite its maturity, amine-based PCCC technology suffers from high operating energy costs, primarily due to the energy required to regenerate the spent amine at elevated temperatures, typically between 120 °C and 140 °C under pressures of 1–2 bar [2,4,5,6,7,8]. Reducing this energy demand has become a major focus of ongoing research [2,3,4,5,6,7,9,10,11]. In recent years, several strategies have been explored to address this challenge. One approach involves replacing conventional steam heating with microwave heating [2,12,13,14,15] or frequency-tuned infrared preferential heating [16] of amine solutions. Another research direction has investigated alternative amine types [17,18,19,20,21,22,23], including monoethanolamine (MEA), diethanolamine (DEA), triethanolamine (TEA), diisopropanolamine (DIPA), 2-amino-2-methyl-1-propanol (AMP), and methyldiethanolamine (MDEA). A third strategy has investigated the use of different solvents to reduce the energy required for amine regeneration, including a wide range of aqueous [24,25,26,27,28] and non-aqueous solvent [28,29,30,31,32,33,34,35,36].

In our previous computational chemistry study [28], we investigated the potential of non-aqueous solutions to reduce the energy penalty associated with amine regeneration in amine-based PCCC technology. We demonstrated that non-aqueous solvents with low dielectric constants, such as diethylene glycol monoethyl ether (DEGMEE), can significantly enhance the energy efficiency of the regeneration process. From a thermodynamic perspective, these non-aqueous solutions exhibit a decrease in regeneration enthalpy, indicating reduced overall energy consumption. In addition, the corresponding reduction in activation energies suggests that CO_2_ desorption could proceed effectively at substantially lower temperatures. Such improvements could enable the use of low-grade or waste heat as the primary regeneration energy source, thereby reducing reliance on high-grade electrical energy.

In the present series of papers, we evaluate the potential of replacing conventional steam heating with frequency-tuned infrared (IR) preferential heating [16] of amine solutions. Excitation of amine solutions using electromagnetic radiation is expected to involve both thermal and non-thermal effects. Non-thermal effects refer to modifications in chemical reaction behaviour arising directly from interactions with the electromagnetic field, rather than from bulk temperature increases. We have previously investigated thermal effects in this context [16], where classical molecular dynamics simulations were used to evaluate frequency-tuned heating of aqueous amine solutions exposed to electromagnetic radiation across the microwave and infrared regions. Those results showed that preferential heating of either water or the amine can be achieved by targeting their respective vibrational frequencies in the infrared range, suggesting that IR heating could provide an efficient route for amine regeneration.

In the present two-paper series, we employ high-accuracy quantum chemical calculations to investigate the non-thermal effects induced under such electromagnetic heating conditions at the molecular level. In this first part of the series, we focus exclusively on the case of external static electric fields, with the objective of establishing a mechanistic and thermodynamic reference framework for the representative CO_2_ absorption–desorption pathways of MEA and TEA. This static field analysis is intended to provide a useful baseline for understanding the intrinsic field sensitivity of the reaction free-energy landscape, including the relative stabilization of reactants, transition states, and products. Importantly, the present results are not intended to directly predict the behaviour under oscillatory infrared laser excitation, which constitutes a time-dependent non-equilibrium perturbation. Rather, they provide a physically interpretable reference point for understanding the possible limitations and mechanistic trends relevant to frequency-tuned infrared excitation of specific vibrational modes, which will be explicitly examined in the second part of this series.

## 2. Computational Details

We start by justifying our selection of the amines and solvent types investigated in this study. The primary amine monoethanolamine (MEA) was chosen because it is the most widely used amine in industrial PCCC applications [14]. We also investigated the tertiary amine triethanolamine (TEA), as it differs fundamentally from MEA in both its reaction mechanism and reaction products. While the reaction between CO_2_ and MEA proceeds via the formation of zwitterion intermediate followed by the formation of carbamate and protonated MEA, the reaction between CO_2_ and TEA produces bicarbonate and protonated TEA in a single reaction step [37]. The contrast between MEA and TEA therefore provides an opportunity to examine how electric fields influence reactions with distinct mechanistic pathways. With respect to the solvent choice, both water and DEGEME were considered for the primary amine MEA, allowing us to explore possible interplay between solvent and static electric field effects.

Quantum computational chemistry calculations were performed using the ORCA 5.0.4 software package [38]. All computations were carried out on the Eddie high-performance computing cluster at the University of Edinburgh. Geometry optimizations and subsequent thermochemical frequency calculations were performed using density functional theory (DFT). Our DFT calculations employed combination of the hybrid meta-GGA TPSS0 functional and the Def2-TZVP triple-zeta valence polarized basis set. This combination of the TPSS0 functional and Def2-TZVP basis set was shown in our previous work to provide the best agreement with available experimental data [28].

It is well established that DFT methods provide reliable geometries and vibrational frequencies for molecular systems. However, electronic energies obtained directly from DFT are generally less accurate, and therefore benefit from refinement using higher-level electronic structure methods. In this work, more accurate electronic energies were computed using the domain-based local pair natural orbital coupled-cluster method, DLPNO-CCSD(T), as implemented in the ORCA package [38]. Single-point DLPNO-CCSD(T) energy calculations were performed on geometries optimized at the DFT level. To account for solvent effects, both the DFT and coupled-cluster calculations employed the polarizable continuum model (PCM) [39] together with Pauling van der Waals atomic radii [40].

A limitation of the present computational framework is the use of an implicit solvent model, which captures the bulk dielectric response of the liquid phase but does not explicitly describe extended solvent networks, solvent-mediated proton transfer pathways, or dynamic hydrogen-bonding rearrangements. The use of fully explicit solvent models within the present high-accuracy DFT framework is not computationally feasible. It should be noted, however, that in the case of TEA reaction, an explicit water molecule was included because it directly participates in the bicarbonate-forming reaction mechanism. Thus, the chemically active water molecule is treated explicitly, while the surrounding bulk solvent environment is represented as a dielectric continuum. To rule out possible electric field artefacts associated with the use of an implicit solvent model, we additionally performed semiempirical computational chemistry simulations with fully explicit solvent models. The resulting relative energetic trends under static electric fields were found to be in very good agreement with the DFT results reported in the present study, providing additional support for the robustness of the reported electric field-induced effects.

In this paragraph, we justify our choice of the above computational details. In our previous computational study on monoethanolamine (MEA) regeneration [28], we rigorously benchmarked our computational framework to reproduce experimental activation energy of zwitterion formation. Geometry optimizations, intrinsic reaction coordinate calculations, and vibrational analyses were performed at the DFT/Def2-TZVP level of theory, with final electronic energies were computed at the DLPNO-CCSD(T)/Def2-TZVP level of theory. A wide range of DFT functionals were systematically evaluated [28]. The TPSS0 DFT functional predicted zwitterion formation activation energy of 11.13 kcal/mol, in an excellent agreement with experimental activation energies (11.14–11.16 kcal/mol) [41,42]. This benchmarking confirmed both the correct reaction mechanism and quantitatively reliable activation barriers, providing strong confidence in the robustness and predictive capability of the computational framework employed in the present study.

In the following paragraphs, we outline the computational workflow used in this study. First, the geometries of all reactants, transition states, and products were optimized using DFT in the absence of any external electric field. After geometry optimization, the centres of mass of all species were translated to the origin at (0,0,0). Single-point energy calculations were then performed on these shifted geometries in order to determine the dipole moment vectors of the reactants, transition states, and products. These dipole moment vectors were subsequently used to define the orientation of the applied static electric fields.

In the following, we justify our choice of the electric field direction as the direction of the total molecular dipole moment vector. In most quantum computational chemistry studies of static electric field effects on chemical reactions, the field is typically aligned along a specific bond or reaction coordinate, because the field component projected along that direction generally produces the strongest catalytic effect. For example, Shaik et al. showed that barrier lowering occurs when the field stabilizes the transition state more than the reactant by acting along the dominant dipole-change direction [43]. Similarly, the formic acid dimer provides a clear illustration: fields applied parallel to the C-C bond substantially reduce the barrier, whereas orthogonal fields have only a minor effect [44]. Related orientation-dependent effects on molecular energies, equilibrium bond geometries, and vibrational Stark shifts under external electric fields have also been systematically demonstrated by Sowlati-Hashjin and Matta [45].

During the development of the present computational protocol, we also examined electric fields applied parallel and antiparallel to the key bond-forming reaction-coordinate directions, namely the N_MEA_-C_CO2_ bond in the case of MEA and the O_OH_-C_CO2_ bond in the case of TEA. These exploratory calculations showed that fields applied along the bond direction (i.e., the direction from the less electronegative atom to the more electronegative atom) stabilize the carbamate product in the case of MEA and the bicarbonate product in the case of TEA, whereas reversing the field direction destabilizes these products. Importantly, these tests demonstrated that applying the field along the bond direction or along the total molecular dipole moment vector leads to the same qualitative stabilization behaviour, although the effect is slightly weaker in the case of dipole alignment.

Based on the above observations, the static field was aligned along the molecular dipole moment vector in the production calculations. This choice maximizes the first-order Stark interaction term (−μ⋅E) and provides a global, structure-dependent direction that reflects the overall charge separation of the system, thereby establishing a consistent mechanistic reference direction across all reactants, transition states, and products. We acknowledge that this represents an idealized reference orientation rather than a complete orientation-averaged description of liquid-phase systems. The purpose of the present work is therefore to establish a physically interpretable baseline for static field effects. This choice is also consistent with prior studies showing that catalytic effects correlate with dipole differences between the reactant and transition state when the external electric field is aligned with a chemically relevant molecular direction [46].

The alignment of the applied static electric field along the molecular dipole moment vector should be regarded as an idealized best-case reference orientation, intended to quantify the intrinsic field sensitivity of the reaction pathways. In real liquid-phase systems, molecular orientations are continuously fluctuating and subject to rotational averaging, which may reduce the net observable field effect. To assess the plausibility of field-induced alignment, the field–dipole interaction energy term (μ·E) was compared with the thermal energy (k_B_T). For the present MEA and TEA systems, the calculated interaction energies for carbamate and bicarbonate range from approximately 1.4–1.5 k_B_T at electric field strength of 0.01 V/Å to 6.8–7.4 k_B_T at electric field strength of 0.05 V/Å, indicating that the applied field is sufficiently strong to bias molecular orientation and partially overcome thermal rotational fluctuations, particularly at higher field strengths.

For oscillating fields (e.g., infrared laser), the situation differs fundamentally. At infrared frequencies, the electric field reverses direction on a femtosecond timescale, which is much faster than molecular rotational motion. As a result, permanent dipoles cannot reorient to follow the instantaneous field direction. Instead, the interaction occurs primarily through molecular polarizability, inducing dipoles that oscillate with the field [47]. Thus, unlike the static field case, where the field direction represents a fixed geometric parameter, infrared laser-molecule coupling depends critically on the field frequency, pulse duration, and molecular rotational dynamics, which together determine the possibility of selectively driving vibrational modes associated with the reaction coordinate.

There are two different approaches for applying an external electric field within the ORCA code [38]. The first approach involves the implicit addition of a perturbative interaction term to the total energy Hamiltonian. The second approach consists of placing the molecular system between two parallel plates carrying opposite point charges. Although the implicit approach is simpler to use, we did not adopt it in this work because it is not available in the ORCA code for transition state geometry optimizations, and ORCA does not account for the applied electric field during frequency calculations. Instead, we explicitly applied static electric fields using parallel plates of point charges, which represents a more physically realistic and fully functional approach for all stages of the calculations.

In this paragraph, we describe the construction of the parallel plates of point charges used to generate the external electric fields. These plates were created using the TITAN electric field generation tool [48], which constructs two circular parallel plates composed of oppositely charged point charges. The separation between the two plates was fixed at 60 Å, with the molecular centre of mass positioned midway between them. Each plate consisted of 30 concentric rings of point charges, with a spacing of 2.0 Å between neighbouring rings. The large plate radius and wide separation ensured the generation of a highly homogeneous electric field within the region containing the molecule. To achieve optimal alignment, the direction of the applied electric field was set parallel to the molecular dipole moment. Each pair of plates contained a total of 5872-point charges, with the magnitude of each point charge determined by the desired electric field strength. During ORCA calculations, the coordinates and magnitudes of the point charges were read from external input files [38]. The correct orientation of the applied electric field was verified by comparing electronic energies and total dipole moments obtained from two sets of single-point energy calculations performed with opposite field directions.

**Figure 1:**
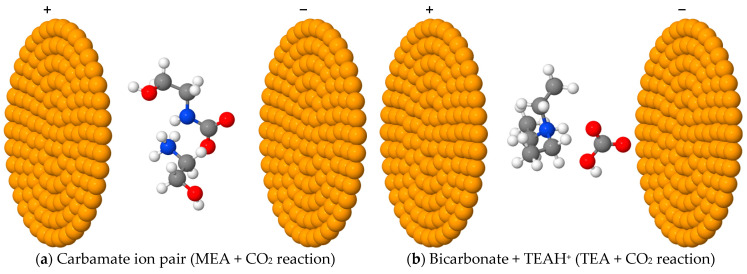
Examples of circular point-charge parallel plates (orange) used to induce an external static electric field. The same setup was used for all other molecular species. The parallel-plate geometries were generated using the TITAN code [48]. To ensure a homogeneous electric field throughout the region between the plates, the actual plate radii and the separation distance are much larger than those depicted in this figure.

Figure 1 illustrates representative configurations of the circular parallel plates of point charges used to generate the external static electric fields applied in this study. It is important to note that the plate size and separation in Figure 1 are reduced for visual clarity. The actual plate radii and the separation between the plates used in the calculations were significantly larger. This design ensures that the molecular species experience an electric field that is effectively uniform across the entire region occupied by the reacting system.

The previously optimized geometries of all reactants, transition states, and products were subsequently reoptimized under external static electric fields with field strengths ranging from 0.0 to 0.05 V/Å. Although these field strengths exceed typical experimental values, they remain well below the ranges commonly employed in computational chemistry studies. Although the static electric field strengths considered in the present study exceed the macroscopic average fields typically encountered in bulk liquid-phase experimental systems, they are employed here to establish a clear mechanistic sensitivity baseline for the response of the reaction free-energy landscape. It is important to note that the local electric field experienced at molecular scale can be substantially larger than the average macroscopic field due to solvent polarization, hydrogen-bond networks, and transient intermolecular dipole configurations. Previous molecular simulation studies of liquid environments have explicitly shown that the local electric field can be significantly larger than the average macroscopic electric field in the liquid phase [49]. In highly polar liquid environments, such local field amplification has also been reported to exceed the externally applied Maxwell field through a local field correction factor greater than unity, with typical values in the range of 1.4–1.8 [50].

Frequency calculations were then performed on all field-optimized geometries at the same level of theory. Single-point coupled-cluster calculations were subsequently carried out on these optimized structures to obtain high-accuracy electronic energies. The resulting electronic energies were combined with the DFT thermochemical data to compute final thermodynamic quantities, including activation energies and reaction enthalpies. Additional details of the computational framework are provided in our recent work [28]. Details on how to compute thermochemical properties, including activation energies and reaction enthalpies, are available elsewhere [51].

## 3. Results and Discussion

### 3.1. Reaction Mechanisms

**Figure 2:**
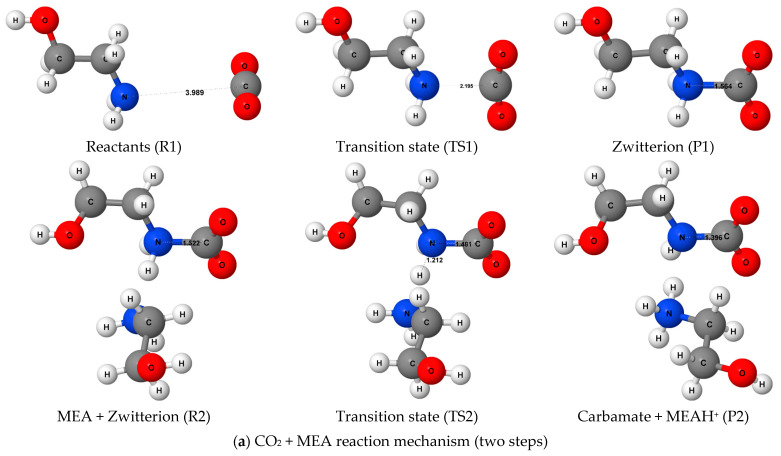

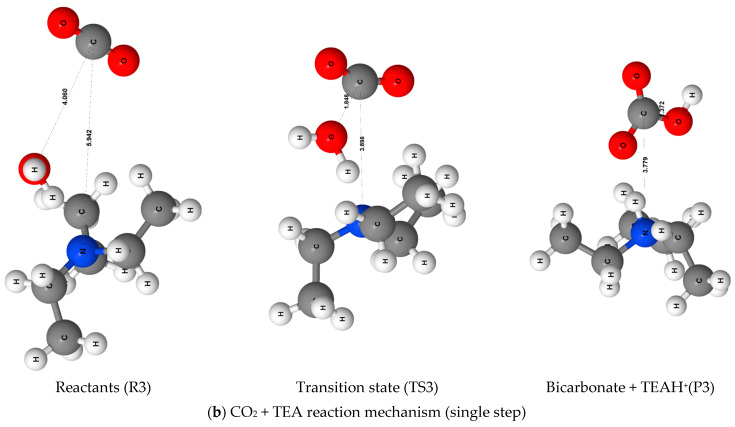
Optimized molecular geometries of the reactants (Rs), transition states (TSs), and products (Ps) involved in the reactions of CO_2_ molecule with the primary amine MEA (**a**) and the tertiary amine TEA (**b**) in the presence of water as solvent. The reported structures were obtained from DFT geometry optimizations performed at the TPSS0/Def2-TZVP level of theory. Bond lengths of key chemical bonds are reported in angstroms.

We begin the Results and Discussion section by describing the chemical reaction mechanisms involved in the absorption of CO_2_ molecules by primary and tertiary amines. Figure 2 presents the optimized geometries of the reactants (Rs), transition states (TSs), and products (Ps) involved in the reactions of CO_2_ molecule with the primary amine MEA (Figure 2a) and the tertiary amine TEA (Figure 2b) in the presence of water as solvent. Although the optimized molecular structures shown in Figure 2 were obtained in the absence of any external electric field, no changes in the underlying reaction mechanisms were observed upon application of the electric field.

As shown in Figure 2a, the reaction between CO_2_ and MEA proceeds through a well-established two-step mechanism. In the first step, a CO_2_ molecule reacts with the nitrogen atom of MEA via a nucleophilic attack, passing through transition state TS1 and forming a zwitterionic intermediate (P1) [28,52,53]. The second step involves intramolecular proton transfer from the zwitterion to a neighbouring MEA molecule, proceeding through transition state TS2 and resulting in the formation of carbamate and protonated MEA (MEAH^+^) (P2). The optimized geometries show a continuous shortening of the C-N bond length as the reaction progresses from the zwitterion to the carbamate species [28,52], which is characteristic of reactions evolving from nucleophilic addition to proton-transfer processes [53].

In contrast to MEA, the reaction between CO_2_ and triethanolamine (TEA) follows a fundamentally different mechanism [37]. Because TEA lacks N-H bonds, carbamate formation is not possible. Instead, in aqueous solution, CO_2_ reacts with TEA through a water-mediated single-step pathway in which a water molecule facilitates simultaneous proton transfer to TEA and formation of bicarbonate. Specifically, a hydroxide group originating from a water molecule attacks the CO_2_ molecule to form bicarbonate, while TEA accepts a proton from the same water molecule, resulting in the formation of protonated TEA (TEAH^+^). In this process, TEA acts as a Brønsted-Lowry base by accepting a proton during CO_2_ hydration. This mechanistic distinction between MEA and TEA provides an important basis for examining how electric fields influence absorption and regeneration reactions in amines with fundamentally different reaction pathways.

### 3.2. Reactions Energy Profiles

**Figure 3:**
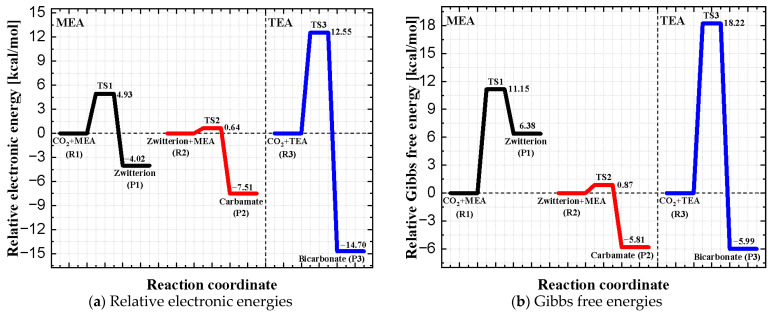
Relative electronic energies (**a**) and Gibbs free energies (**b**) corresponding to the optimized geometries shown in Figure 2. Electronic energies were computed using the coupled-cluster method at the DLPNO-CCSD(T)/Def2-TZVP level of theory. Gibbs free energies were obtained by combining the coupled-cluster electronic energies with DFT frequency calculations performed at the TPSS0/Def2-TZVP level of theory. In all cases, the energies of the reactants were taken as the reference baseline.

Relative electronic energies and Gibbs free energies corresponding to the reactants (Rs), transition states (TSs), and products (Ps) shown in Figure 2 are presented in Figure 3. Electronic energies describe the intrinsic stability of molecular structures at 0 K, whereas Gibbs free energies additionally incorporate thermal contributions and are therefore more appropriate for describing thermodynamic stability under experimental conditions. In practice, Gibbs free energies are the key quantities used to evaluate reaction activation energies. The relative electronic energies reported in Figure 3a were computed using the coupled-cluster method, while the Gibbs free energies shown in Figure 3b were obtained by combining coupled-cluster electronic energies with DFT frequency calculations. In all cases, the energies of the reactants were taken as the reference baseline.

For the case of MEA, both the electronic energy and Gibbs free energy profiles confirm the well-known two-step reaction mechanism involving zwitterion formation followed by carbamate formation. As shown in Figure 3b, the zwitterion intermediate is stabilized relative to transition state TS1 but remains higher in Gibbs free energy than the reactants, consistent with its transient nature [53] and the experimental difficulty associated with its direct detection. Figure 3b further shows that regeneration of the zwitterion from the carbamate species involves an activation energy of 6.68 kcal/mol, whereas regeneration of MEA from the zwitterion requires an activation energy of 4.77 kcal/mol. Consequently, regeneration of the zwitterion from carbamate constitutes the rate-limiting step in the MEA regeneration process [54,55].

As shown in Figure 3, CO_2_ absorption by TEA differs markedly from the case of MEA. Because TEA cannot form carbamate species, its reaction with CO_2_ proceeds through a single transition state (TS3), leading directly to the formation of bicarbonate and protonated TEA (P3). According to Figure 3a, the activation energy for CO_2_ absorption in the case of TEA is approximately 18.22 kcal/mol, compared to 11.15 kcal/mol for MEA, indicating significantly slower absorption kinetics in the case of TEA. With respect to amine regeneration, the activation energy is 24.21 kcal/mol for TEA, compared to 6.68 kcal/mol for MEA. These results indicate that regeneration of TEA is substantially more challenging and would require much higher regeneration temperatures than regeneration of MEA.

### 3.3. Effect of Electric Field on CO_2_ Absorption

**Figure 4:**
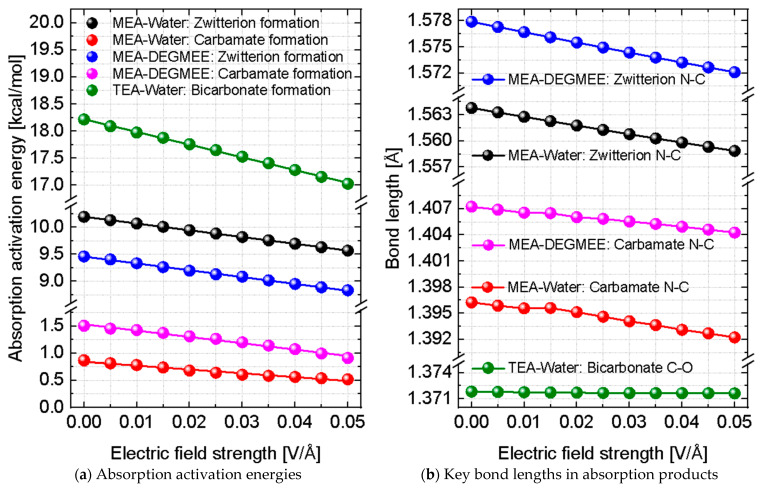
Effects of static electric field strength on the absorption of CO_2_ by primary (MEA) and tertiary (TEA) amines. (**a**) Activation energies for zwitterion and carbamate formation in the case of MEA, and for bicarbonate formation in the case of TEA. (**b**) Key bond lengths in the absorption products, including the N_MEA_-C_CO2_ bonds in the zwitterion and carbamate species, and the O_Water_-C_CO2_ bond in bicarbonate. Water was used as solvent in all cases, while DEGEME was considered only for MEA.

In this section, we report and discuss the non-thermal effects induced by an external static electric field on the CO_2_ absorption process. Figure 4 illustrates how the CO_2_ absorption activation energies (left) and key bond lengths (right) vary as a function of the applied static electric field strength for both MEA and TEA. Clear and systematic trends are observed in both activation energies and bond lengths, revealing pronounced non-thermal effects of the electric field. Increasing the electric field strength leads to a reduction in absorption activation energies and a shortening of the bonds formed between the CO_2_ molecule and the amine, indicating enhanced absorption kinetics and increased stabilization of the absorption products. Regardless of the electric field strength, the activation energies associated with zwitterion formation are significantly lower in the case of DEGEME, reflecting faster absorption kinetics in this solvent. In contrast, the activation energy for carbamate formation is higher in DEGEME, consistent with our previous DFT study on solvent effects [28].

Table 1 reports the relative changes in the activation energies for zwitterion and bicarbonate formation, together with the associated CO_2_–amine bond lengths, as a function of the applied electric field strength. This table quantifies both the magnitude of the non-thermal electric field effects and the role of amine and solvent type. For all systems considered, the absorption activation energies decrease as the electric field strength increases, accompanied by a shortening of the corresponding N-C bonds in the reaction products. These trends indicate faster CO_2_ absorption kinetics and increased stabilization of the absorption products under an applied electric field. The activation energies decrease by approximately 6% at the highest field strength considered, and this effect appears to be largely independent of amine type and solvent. In the case of MEA, the reduction in the N_MEA_-C_CO2_ bond length is slightly larger when DEGEME is used as the solvent, suggesting a marginally stronger field effect. By contrast, in the case of TEA, the O_Water_-C_CO2_ bond length remains nearly constant with increasing field strength, reflecting the fact that the reaction is mediated primarily by the solvent rather than by direct bonding to the amine.

### 3.4. Effects of Electric Field on CO_2_ Desorption

**Figure 5:**
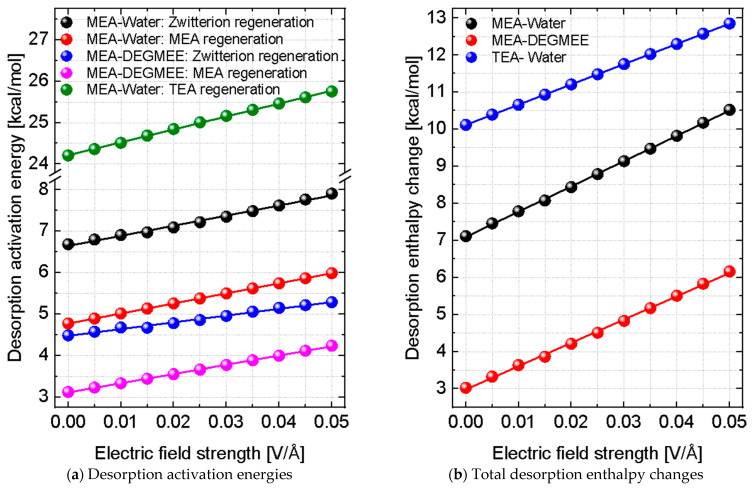
Effect of static electric field strength on the desorption of CO_2_ from primary (MEA) and tertiary (TEA) amines in the presence of water or DEGEME as solvent. (**a**) Activation energies associated with the rate-limiting regeneration steps for MEA and TEA. (**b**) Total enthalpy changes associated with regeneration of MEA and TEA.

The most important results of this study concern the non-thermal effects of an external static electric field on amine regeneration reactions. Figure 5 shows the influence of static electric fields on the desorption (regeneration) of CO_2_ from MEA and TEA, expressed in terms of the corresponding regeneration activation energies (Figure 5a) and total enthalpy changes (Figure 5b). An increase in regeneration activation energy indicates slower regeneration kinetics and a requirement for higher regeneration temperatures, while an increase in the total regeneration enthalpy reflects higher overall energy consumption during the amine regeneration process.

We first discuss the results obtained in the absence of any applied electric field, where the effects of both solvent and amine type are particularly pronounced. In the case of MEA, regeneration of the zwitterion intermediate constitutes the rate-limiting step, and the results clearly show that the use of DEGEME as a solvent with low dielectric constant (12.6) would enable faster regeneration and lower regeneration temperature requirements compared to water as a solvent with large dielectric constant (78.36). These results agree very well with our previous DFT study on effect of solvent dielectric constant on the regeneration process [28].

The effect of amine type is also significant. Regeneration of TEA is considerably more challenging than regeneration of MEA, involving substantially higher activation energies. Similar trends are observed for the regeneration enthalpy. For MEA, replacing water with DEGEME leads to a reduction in the energy required for regeneration, whereas systems containing TEA require a higher overall heat input to achieve amine regeneration.

The results shown in Figure 5 reveal a striking contrast with the behaviour observed for CO_2_ absorption. Whereas the application of static electric field consistently lowers the activation energies for CO_2_ absorption, it leads to an increase in the energy barriers associated with CO_2_ desorption. This indicates that static electric fields stabilize the CO_2_–amine reaction products, thereby hindering the regeneration reactions.

For all systems studied, the desorption enthalpy increases monotonically with increasing electric field strength, demonstrating that stronger fields make CO_2_ release progressively more endothermic. The relative changes in the regeneration activation energies and reaction enthalpies for MEA and TEA are summarized in Table 2. The presence of an electric field therefore leads to slower regeneration kinetics and increased temperature requirements.

The detrimental effect of the static electric field on regeneration is considerably stronger in the case of MEA than in the case of TEA. Application of a static electric field with a strength of 0.05 V/Å increases the regeneration activation energy by approximately 18.3% for MEA, compared to an increase of about 6.4% for TEA. A similar trend is observed for the regeneration enthalpy. As shown in Table 2, systems containing MEA experience a much larger increase in heat consumption under an applied electric field than systems containing TEA. This effect becomes particularly pronounced when water is replaced by DEGEME, where the regeneration enthalpy increases by more than 100% at the highest field strength considered. In our previous study [28], we have shown that using solvents with low dielectric constants, such as DEGMEE, is advantageous since it reduces regeneration activation energy and enthalpy. From the present study we can see that the presence of electric field can compromise this advantage. This demonstrates a strong interplay between the external electric field and solvent effects.

The effect of the applied static electric field on the regeneration process can be analyzed from both kinetic and thermodynamic perspectives [28]. In the present study, the reaction kinetics is represented by the regeneration activation energy, while the reaction thermodynamics is described by the regeneration enthalpy change. The observed increase in regeneration activation energy under the applied static electric field indicates a decrease in regeneration efficiency, since the rate-limiting desorption step becomes kinetically less accessible. As a result, higher regeneration temperatures would be required to achieve the same regeneration rate. In parallel, the increase in regeneration enthalpy reflects a higher thermal energy consumption for the amine regeneration process. Therefore, the combined increase in both the regeneration activation energy and enthalpy demonstrates that the applied electric field adversely affects the thermal regeneration performance and increases the overall energy consumption of the process.

Overall, the results presented in Figure 5 and Table 2 demonstrate that external static electric fields significantly hinder the regeneration of both MEA and TEA, with MEA being disproportionately affected due to its carbamate-forming reaction pathway. Furthermore, the situation of MEA becomes even worse with the use of solvents with low dielectric constants such as DEGMEE. These findings will probably have important implications for frequency-tuned heating strategies, as it indicates that non-thermal stabilization induced by electric fields can counteract the fundamental requirements for efficient amine regeneration. Any potential benefits associated with electromagnetic heating must therefore be carefully balanced against these adverse non-thermal effects in order to avoid inadvertently increasing the energy penalty associated with CO_2_ release.

To provide a more rigorous theoretical basis for the observed increase in regeneration barriers, we performed a first-order Stark analysis. Under our computational protocol, the external electric field for each stationary point is aligned with the corresponding total dipole moment vector. Therefore, the field vectors used for the product and transition state are state-specific and are denoted as $E_{P}$ and $E_{TS}$, respectively. According to the first-order Stark expansion, the electronic contribution to the regeneration activation energy as a function of the applied field can be calculated from Equation (1), where $E_{P}(0)$ and $E_{TS}(0)$, are the zero-field electronic energies of the product and transition state, and $\mu_{P}\left(0\right)$ and $\mu_{TS}\left(0\right)$ are their corresponding zero-field dipole moment vectors.

(1) $$RE(E)\;=\left[E_{TS}\;(0)-E_{P}(0)\right]-\left[(\mu_{TS}(0)\cdot E_{TS})-({\mu_{P}(0).E}_{P})\right]$$

The values of $E_{P}(0)$, $E_{TS}(0)$, $\mu_{P}\left(0\right)$ and $\mu_{TS}\left(0\right)$ were taken from DFT calculations in the absence of any electric field. The theoretical regeneration energies predicted by Equation (1) are compared with these fully obtained from DFT calculations in Table 3. The excellent agreement observed for both MEA and TEA over the entire range of electric field strength confirms that the increase in regeneration energy is predominantly governed by the first-order dipole–field interaction term, while higher-order Stark contributions remain comparatively small. Furthermore, this good agreement between theory and DFT calculations further enhances our confidence in the employed computational chemistry framework.

The present study is intended to establish a mechanistic baseline under static external electric fields rather than to directly model laser-assisted amine regeneration. By analyzing the response of CO_2_–amine systems to static electric fields, we isolate how external fields reshape the free-energy landscape, stabilize absorption products, and modify regeneration energetics in the absence of time-dependent, non-equilibrium, or mode-selective effects. This approach enables identification of the reaction steps that are intrinsically sensitive to field-matter interactions under static field conditions and provides a well-defined reference framework for subsequent dynamic investigations.

It is important to emphasize that the present work does not make any direct quantitative claims regarding infrared (IR) laser-assisted regeneration. Static electric fields primarily perturb the thermodynamic landscape through sustained stabilization or destabilization of stationary points on the free-energy surface. In contrast, oscillatory IR laser excitation [56] constitutes a fundamentally time-dependent, non-equilibrium perturbation involving frequency selectivity, possible vibrational resonance, and dynamical coupling to nuclear motion. The explicit analysis of these effects lies beyond the scope of the present paper and will be addressed in Part II of this series, where IR laser excitation at different vibrational frequencies will be examined directly using the static field results reported here solely as a mechanistic baseline.

## 4. Conclusions

In this work, we conducted high-accuracy quantum computational chemistry calculations to investigate the non-thermal effects of external static electric fields on CO_2_ absorption and desorption reactions in amine-based post-combustion carbon capture systems. By examining both primary (MEA) and tertiary (TEA) amines under aqueous and non-aqueous conditions, this study was designed to establish a mechanistic reference framework that isolates intrinsic electric field effects on reaction energetics, independent of thermal or time-dependent excitation.

By quantifying how static electric fields reshape the mechanistic and thermodynamic landscape of CO_2_–amine reactions, this study defines clear intrinsic limits for field-assisted carbon capture strategies. The results show that non-thermal stabilization induced by static electric fields systematically opposes low-energy amine regeneration, particularly for carbamate-forming systems such as MEA. These findings establish an essential mechanistic baseline against which more complex, time-dependent excitation schemes must be evaluated. Future developments in infrared laser-assisted carbon capture must therefore rely on carefully selected excitation frequencies and non-equilibrium driving mechanisms capable of overcoming, rather than reinforcing, the static field stabilization effects identified here. In contrast to static electric fields, frequency-tuned infrared laser excitation can selectively drive vibrational modes along the reaction coordinate, potentially enabling non-thermal lowering of the amine regeneration barrier. These possibilities will be investigated in Part II of this work.

## Figures and Tables

**Table 1:** Non-thermal effects of static electric field on CO_2_ absorption reactions. Relative changes in the activation energies for zwitterion and bicarbonate formation and the corresponding changes in CO_2_–amine bond lengths as a function of electric field strength. The labels **W** and **D** in **MEA-W**, **MEA-D**, and **TEA-W** refer to water and DEGMEE, respectively.

E [V/Å]	Activation Energy Change [%]			Bond Length Change [%]		
	MEA–W	MEA-D	TEA–W	MEA–W N_MEA_-C_CO2_	MEA-D N_MEA_-C_CO2_	TEA–W O_Water_-C_CO2_
0.0	0.0	0.0	0.0	0.0	0.0	0.0
0.005	−0.61	−0.58	−0.69	−0.03	−0.04	−0.0025
0.010	−1.21	−1.34	−1.32	−0.07	−0.08	−0.0049
0.015	−1.82	−2.08	−1.86	−0.10	−0.11	−0.0070
0.020	−2.43	−2.78	−2.51	−0.13	−0.15	−0.0087
0.025	−3.04	−3.49	−3.11	−0.16	−0.19	−0.0104
0.030	−3.65	−3.96	−3.78	−0.19	−0.22	−0.0116
0.035	−4.27	−4.67	−4.44	−0.22	−0.26	−0.0126
0.040	−4.88	−5.39	−5.12	−0.25	−0.30	−0.0131
0.045	−5.50	−6.02	−5.82	−0.29	−0.33	−0.0138
0.050	−6.13	−6.61	−6.52	−0.32	−0.37	−0.0144

**Table 2:** Non-thermal effects of a static electric field on amine regeneration reactions. Relative changes in the activation energies of the rate-limiting regeneration steps and in the total regeneration enthalpy as a function of electric field strength. The labels **W** and **D** in **MEA-W**, **MEA-D**, and **TEA-W** refer to water and DEGMEE, respectively.

E [V/Å]	Activation Energy Change [%]			Enthalpy Change [%]		
	MEA–W	MEA-D	TEA–W	MEA–W	MEA-D	TEA–W
0.0	0.0	0.0	0.0	0.0	0.0	0.0
0.005	1.67	1.98	0.63	4.84	9.94	2.75
0.010	3.31	4.25	1.25	9.42	20.03	5.37
0.015	4.27	4.09	1.97	13.53	27.46	8.07
0.020	6.04	6.56	2.63	18.58	38.96	10.81
0.025	7.94	8.26	3.30	23.50	48.80	13.50
0.030	9.91	10.47	3.94	28.43	59.39	16.23
0.035	11.92	12.81	4.54	33.21	70.72	18.88
0.040	13.97	14.85	5.17	38.03	81.80	21.58
0.045	16.15	16.28	5.79	43.01	92.50	24.34
0.050	18.28	17.86	6.38	47.89	103.41	27.03

**Table 3:** Theoretical electronic regeneration energies obtained from the first-order Stark expansion (Equation (1)) compared with these fully obtained from DFT calculations for the cases of MEA and TEA at different applied electric field strengths.

Electric Field Strength $\left|E\right|$ [V/Å]	MEA Electronic Regeneration Energy [kcal/mol]		TEA Electronic Regeneration Energy [kcal/mol]	
	Theory	DFT	Theory	DFT
0.0	9.162	9.162	28.363	28.363
0.005	9.293	9.287	28.533	28.614
0.010	9.424	9.413	28.703	28.740
0.015	9.555	9.538	28.872	28.928
0.020	9.687	9.664	29.042	29.116
0.025	9.818	9.789	29.211	29.305
0.030	9.949	9.978	29.381	29.430
0.035	10.080	10.103	29.550	29.619
0.040	10.211	10.291	29.720	29.870
0.045	10.342	10.417	29.889	29.995
0.050	10.474	10.605	30.059	30.183

## Data Availability

Dataset available on request from the corresponding author.

## References

[1] Liang Z., Rongwong W., Liu H., Fu K., Gao H., Cao F., Zhang R., Sema T., Henni A., Sumon K. (2015). Recent Progress and New Developments in Post-Combustion Carbon-Capture Technology with Amine Based Solvents. Int. J. Greenh. Gas Control.

[2] McGurk S.J., Martín C.F., Brandani S., Sweatman M.B., Fan X. (2017). Microwave Swing Regeneration of Aqueous Monoethanolamine for Post-Combustion CO_2_ Capture. Appl. Energy.

[3] Rochelle G.T. (2009). Amine Scrubbing for CO_2_ Capture. Science.

[4] House K.Z., Harvey C.F., Aziz M.J., Schrag D.P. (2009). The Energy Penalty of Post-Combustion CO_2_ Capture & Storage and Its Implications for Retrofitting the U.S. Installed Base. Energy Environ. Sci..

[5] Husebye J., Brunsvold A.L., Roussanaly S., Zhang X. (2012). Techno Economic Evaluation of Amine Based CO_2_ Capture: Impact of CO_2_ Concentration and Steam Supply. Energy Procedia.

[6] Dutcher B., Fan M., Russell A.G. (2015). Amine-Based CO_2_ Capture Technology Development from the Beginning of 2013-A Review. ACS Appl. Mater. Interfaces.

[7] Yu C.-H., Huang C.-H., Tan C.-S. (2012). A Review of CO_2_ Capture by Absorption and Adsorption. Aerosol. Air Qual. Res..

[8] He X., He H., Barzagli F., Amer M.W., Li C., Zhang R. (2023). Analysis of the Energy Consumption in Solvent Regeneration Processes Using Binary Amine Blends for CO_2_ Capture. Energy.

[9] Bougie F., Pokras D., Fan X. (2019). Novel Non-Aqueous MEA Solutions for CO_2_ Capture. Int. J. Greenh. Gas Control.

[10] Chen Y.-S., Chiu H.-H., Jao H.-S., Kiew Y.-Q., Yu B.-Y. (2025). Progress in Modeling of Carbon Capture Technologies. Cambridge Prism. Carbon Technol..

[11] Du J., Yang W., Xu L., Bei L., Lei S., Li W., Liu H., Wang B., Sun L. (2024). Review on Post-Combustion CO_2_ Capture by Amine Blended Solvents and Aqueous Ammonia. Chem. Eng. J..

[12] Afify N.D., Sweatman M.B. (2018). Molecular Dynamics Simulation of Microwave Heating of Liquid Monoethanolamine (MEA): An Evaluation of Existing Force Fields. J. Chem. Phys..

[13] Afify N.D., Sweatman M.B. (2018). Classical Molecular Dynamics Simulation of Microwave Heating of Liquids: The Case of Water. J. Chem. Phys..

[14] Bougie F., Fan X. (2018). Microwave Regeneration of Monoethanolamine Aqueous Solutions Used for CO2 Capture. Int. J. Greenh. Gas Control.

[15] Nokpho P., Amornsin P., Boonmatoon P., Wang X., Chalermsinsuwan B. (2024). Evaluating Regeneration Performance of Amine Functionalized Solid Sorbents for Direct Air CO_2_ Capture Using Microwave. Mater. Today Sustain..

[16] Afify N.D., Sweatman M.B. (2019). Preferential Heating of Aqueous Amine Solutions Using Infrared Radiation at Selected Vibrational Frequencies: A Molecular Dynamics Study. J. Chem. Phys..

[17] Rochelle G., Chen E., Freeman S., Van Wagener D., Xu Q., Voice A. (2011). Aqueous Piperazine as the New Standard for CO_2_ Capture Technology. Chem. Eng. J..

[18] Martin S., Lepaumier H., Picq D., Kittel J., de Bruin T., Faraj A., Carrette P.-L. (2012). New Amines for CO_2_ Capture. IV. Degradation, Corrosion, and Quantitative Structure Property Relationship Model. Ind. Eng. Chem. Res..

[19] Hartono A., Ciftja A.F., Brúder P., Svendsen H.F. (2014). Characterization of Amine-Impregnated Adsorbent for CCS Post Combustion. Energy Procedia.

[20] Gjernes E., Helgesen L.I., Maree Y. (2013). Health and Environmental Impact of Amine Based Post Combustion CO_2_ Capture. Energy Procedia.

[21] Closmann F., Nguyen T., Rochelle G.T. (2009). MDEA/Piperazine as a Solvent for CO_2_ Capture. Energy Procedia.

[22] Bougie F., Iliuta M.C. (2010). CO_2_ Absorption into Mixed Aqueous Solutions of 2-Amino-2-Hydroxymethyl-1,3-Propanediol and Piperazine. Ind. Eng. Chem. Res..

[23] Amirsadat S.A., Azari A., Valizadeh A. (2026). Advances in Amine-Based Absorption Solvent Engineering: Co-Solvent Strategies toward Low-Energy Post-Combustion CO_2_ Capture. Results Eng..

[24] Zhao B., Sun Y., Yuan Y., Gao J., Wang S., Zhuo Y., Chen C. (2011). Study on Corrosion in CO_2_ Chemical Absorption Process Using Amine Solution. Energy Procedia.

[25] Park S.-W., Lee J.-W., Choi B.-S., Lee J.-W. (2006). Absorption of Carbon Dioxide into Non-Aqueous Solutions Of N-Methyldiethanolamine. Korean J. Chem. Eng..

[26] El Hadri N., Quang D.V., Goetheer E.L.V., Abu Zahra M.R.M. (2017). Aqueous Amine Solution Characterization for Post-Combustion CO_2_ Capture Process. Appl. Energy.

[27] Lail M., Tanthana J., Coleman L. (2014). Non-Aqueous Solvent (NAS) CO_2_ Capture Process. Energy Procedia.

[28] Afify N.D., Sweatman M.B. (2024). Solvent-Mediated Modification of Thermodynamics and Kinetics of Monoethanolamine Regeneration Reaction in Amine-Stripping Carbon Capture: Computational Chemistry Study. J. Chem. Phys..

[29] Tao M., Gao J., Zhang P., Zhang W., Liu Q., He Y., Shi Y. (2017). Biogas Upgrading by Capturing CO_2_ in Non-Aqueous Phase-Changing Diamine Solutions. Energy Fuels.

[30] Tamajón F.J., Álvarez E., Cerdeira F., Gómez-Díaz D. (2016). CO_2_ Absorption into N-Methyldiethanolamine Aqueous-Organic Solvents. Chem. Eng. J..

[31] Mathias P.M., Zheng F., Heldebrant D.J., Zwoster A., Whyatt G., Freeman C.M., Bearden M.D., Koech P. (2015). Measuring the Absorption Rate of CO_2_ in Nonaqueous CO_2_-Binding Organic Liquid Solvents with a Wetted-Wall Apparatus. ChemSusChem.

[32] Hasib-ur-Rahman M., Larachi F. (2012). CO_2_ Capture in Alkanolamine-RTIL Blends via Carbamate Crystallization: Route to Efficient Regeneration. Environ. Sci. Technol..

[33] Hart R., Pollet P., Hahne D.J., John E., Llopis-Mestre V., Blasucci V., Huttenhower H., Leitner W., Eckert C.A., Liotta C.L. (2010). Benign Coupling of Reactions and Separations with Reversible Ionic Liquids. Tetrahedron.

[34] Cui M., Chen S., Qi T., Zhang Y. (2018). Investigation of CO_2_ Capture in Nonaqueous Ethylethanolamine Solution Mixed with Porous Solids. J. Chem. Eng. Data.

[35] Barzagli F., Lai S., Mani F. (2014). Novel Non-Aqueous Amine Solvents for Reversible CO_2_ Capture. Energy Procedia.

[36] Barzagli F., Mani F., Peruzzini M. (2013). Efficient CO_2_ Absorption and Low Temperature Desorption with Non-Aqueous Solvents Based on 2-Amino-2-Methyl-1-Propanol (AMP). Int. J. Greenh. Gas Control.

[37] Pornjariyawatch C., Jitchum V., Assawatwikrai K., Leepakorn P., Probst M., Boekfa B., Maihom T., Limtrakul J. (2025). Computational Study of Carbon Dioxide Capture by Tertiary Amines. ChemPhysChem.

[38] Neese F., Wennmohs F., Becker U., Riplinger C. (2020). The ORCA Quantum Chemistry Program Package. J. Chem. Phys..

[39] Barone V., Cossi M. (1998). Quantum Calculation of Molecular Energies and Energy Gradients in Solution by a Conductor Solvent Model. J. Phys. Chem. A.

[40] Davran-Candan T. (2014). DFT Modeling of CO_2_ Interaction with Various Aqueous Amine Structures. J. Phys. Chem. A.

[41] Ali S.H. (2005). Kinetics of the Reaction of Carbon Dioxide with Blends of Amines in Aqueous Media Using the Stopped-flow Technique. Int. J. Chem. Kinet..

[42] Alper E. (1990). Reaction Mechanism and Kinetics of Aqueous Solutions of 2-Amino-2-Methyl-1-Propanol and Carbon Dioxide. Ind. Eng. Chem. Res..

[43] Shaik S., Mandal D., Ramanan R. (2016). Oriented Electric Fields as Future Smart Reagents in Chemistry. Nat. Chem..

[44] Arabi A.A., Matta C.F. (2011). Effects of External Electric Fields on Double Proton Transfer Kinetics in the Formic Acid Dimer. Phys. Chem. Chem. Phys..

[45] Sowlati-Hashjin S., Matta C.F. (2013). The Chemical Bond in External Electric Fields: Energies, Geometries, and Vibrational Stark Shifts of Diatomic Molecules. J. Chem. Phys..

[46] Dittner M., Hartke B. (2020). Globally Optimal Catalytic Fields for a Diels–Alder Reaction. J. Chem. Phys..

[47] Zalden P., Song L., Wu X., Huang H., Ahr F., Mücke O.D., Reichert J., Thorwart M., Mishra P.K., Welsch R. (2018). Molecular Polarizability Anisotropy of Liquid Water Revealed by Terahertz-Induced Transient Orientation. Nat. Commun..

[48] Stuyver T., Huang J., Mallick D., Danovich D., Shaik S. (2020). TITAN: A Code for Modeling and Generating Electric Fields—Features and Applications to Enzymatic Reactivity. J. Comput. Chem..

[49] Raiteri P., Kraus P., Gale J.D. (2020). Molecular Dynamics Simulations of Liquid–Liquid Interfaces in an Electric Field: The Water–1,2-Dichloroethane Interface. J. Chem. Phys..

[50] Fried S.D., Wang L.-P., Boxer S.G., Ren P., Pande V.S. (2013). Calculations of the Electric Fields in Liquid Solutions. J. Phys. Chem. B.

[51] Leszczynski J., Kaczmarek-Kedziera A., Puzyn T., Papadopoulos M.G., Reis H., Shukla M.K., Leszczynski J., Kaczmarek-Kedziera A., Puzyn T., Papadopoulos M.G., Reis H., Shukla M.K. (2017). Handbook of Computational Chemistry.

[52] Ben Said R., Kolle J.M., Essalah K., Tangour B., Sayari A. (2020). A Unified Approach to CO_2_ –Amine Reaction Mechanisms. ACS Omega.

[53] Versteeg G.F., van Swaaij W.P.M. (1988). On the Kinetics between CO_2_ and Alkanolamines Both in Aqueous and Non-Aqueous Solutions—I. Primary and Secondary Amines. Chem. Eng. Sci..

[54] Danckwerts P.V. (1979). The Reaction of CO_2_ with Ethanolamines. Chem. Eng. Sci..

[55] Caplow M. (1968). Kinetics of Carbamate Formation and Breakdown. J. Am. Chem. Soc..

[56] Stapelfeldt H., Seideman T. (2003). Colloquium: Aligning Molecules with Strong Laser Pulses. Rev. Mod. Phys..

